# Transformation of Litchi Pericarp-Derived Condensed Tannin with *Aspergillus awamori*

**DOI:** 10.3390/ijms17071067

**Published:** 2016-07-12

**Authors:** Sen Lin, Qing Li, Bao Yang, Xuewu Duan, Mingwei Zhang, John Shi, Yueming Jiang

**Affiliations:** 1 Key Laboratory of Plant Resource Conservation and Sustainable Utilization/Guangdong Provincial Key Laboratory of Applied Botany, South China Botanical Garden, Chinese Academy of Sciences, Guangzhou 510650, China; youhulinsen@hotmail.com (S.L.); yangbao@scbg.ac.cn (B.Y.); xwduan@scbg.ac.cn (X.D.); 2 Wenzhou Institute of Biomaterials and Engineering (in Preparation), Chinese Academy of Science, Wenzhou 325000, China; liqing@wibe.ac.cn; 3 Sericultural & Agri-Food Research Institute, Guangdong Academy of Agricultural Sciences/Key Laboratory of Functional Foods, Ministry of Agriculture/Guangdong Key Laboratory of Agricultural Products Processing, Guangzhou 510610, China; mwzhh@163.net; 4 Guelph Research and Development Center, Agriculture and Agri-Food Canada, Guelph, ON N1G 5C9, Canada; john.shi@agr.gc.ca

**Keywords:** transformation, condensed tannin, *A. awamori*, litchi pericarp, antioxidant activity

## Abstract

Condensed tannin is a ubiquitous polyphenol in plants that possesses substantial antioxidant capacity. In this study, we have investigated the polyphenol extraction recovery and 2,2-diphenyl-1-picrylhydrazyl (DPPH) scavenging activity of the extracted polyphenol after litchi pericarp is treated with *Aspergillus awamori*, *Aspergillus sojae* or *Aspergillus oryzae*. We have further explored the activity of *A. awamori* in the formation of condensed tannin. The treatment of *A. awamori* appeared to produce the highest antioxidant activity of polyphenol from litchi pericarp. Further studies suggested that the treatment of *A. awamori* releases the non-extractable condensed tannin from cell walls of litchi pericarp. The total extractable tannin in the litchi pericarp residue after a six-time extraction with 60% ethanol increased from 199.92 ± 14.47–318.38 ± 7.59 μg/g dry weight (DW) after the treatment of *A. awamori*. The ESI-TOF-MS and HPLC-MS^2^ analyses further revealed that treatment of *A. awamori* degraded B-type condensed tannin (condensed flavan-3-ol via C4–C8 linkage), but exhibited a limited capacity to degrade the condensed tannin containing A-type linkage subunits (C4–C8 coupled C2–O–C7 linkage). These results suggest that the treatment of *A. awamori* can significantly improve the production of condensed tannin from litchi pericarp.

## 1. Introduction

Litchi (*Litchi chinensis* Sonn.), as a subtropical to tropical fruit with high commercial value, is widely planted in south China and Southeast Asian countries, as well as in African countries. It has attractive red-colored pericarp tissue that contains a significant amount of polyphenols [1]. Polyphenols have strong antioxidant activity, inhibit glucosidase and acetylcholinesterase [2], increase lifespan and stress resistance [3], block acetyl cholinesterase [2], alleviate the depressant effect [4] and reduce glucose-induced oxidative cell damage and dysfunction in pancreatic cell [5]. Previously, epicatechin, procyanidin A2, condensed tannin, cyanidin-3-rutinoside and rutin have been identified as the major polyphenols in litchi pericarp [6]. Solvent extraction of polyphenols from litchi pericarp has often been used, and a large amount of polyphenols (up to 50%) remain with the litchi pericarp [7,8]. These non-extractable polyphenols are often conjugated to cell walls with a glycosidic bond or other interactions, making it hard to extract simply using aqueous organic solvent. It has been reported that enzymatic treatments followed by aqueous organic solvent extraction increase the yield of polyphenols from litchi pericarp by releasing non-extractable polyphenols [9,10]. However, it is difficult to choose appropriate enzymes for releasing polyphenols from cell walls, because the enzymes have their specificity for conjugated bonds. Furthermore, the enzymatic activity may decrease in the process, which would greatly limit the application. Using microorganisms instead of enzymes has several advantages: (1) the production of enzymes can be induced in microorganisms that contain multiple enzymes for degrading various substrates; (2) it is an economic way to process plant-derived wastes; and (3) microorganism can sustainably produce the enzymes to release the conjugated polyphenols from cell walls.

A genus of microorganisms, *Aspergillus*, contains various hydrolases and phenolic-related metabolic enzymes for releasing non-extractable polyphenols and transforms these polyphenols. *Aspergillus awamori*, *Aspergillus sojae* or *Aspergillus oryzae* are widely used in producing traditional foods in East Asia and Southeast Asia and are generally perceived as safe microorganisms. Many health benefits from the polyphenol-enriched fermenting foods have been reported [11,12,13,14]. In our previous study, we reported that transformation of the litchi pericarp-derived flavone [15,16] and polysaccharide [17] with the *A. awamori* treatment greatly improved the bioactivities of the litchi pericarp. Here, we have compared *A. awamori*, *A. sojae* and *A. oryzae* for their activity in releasing polyphenols from litchi pericarp. The effects of the *A. awamori* treatment on litchi pericarp-derived condensed tannins were further investigated using electrospray ionization-time of flight-mass spectrometry (ESI-TOF-MS) and high performance liquid chromatography electrospray-mass spectrometry (HPLC-MS).

## 2. Results and Discussion

### 2.1. Effect of Aspergillus Treatments on Polyphenol Recovery and DPPH Radical Scavenging Activity

**Figure 1:**
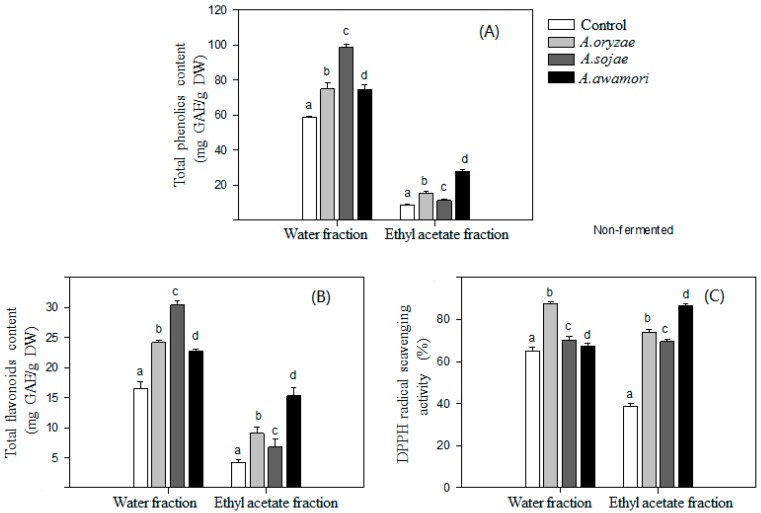
Changes of the contents of total phenolics (**A**) and flavonoids (**B**) with *Aspergillus* treatment and their DPPH radical scavenging activities (**C**). Data marked with the same letter above columns were not significant difference at *p* < 0.05 (mean ± SD, *n* = 3).

The effects of the *Aspergillus* treatment on the polyphenol extraction recovery from litchi pericarp and the antioxidant activity of the extracted polyphenol are shown in Figure 1. The polyphenol extraction recovery with aqueous-organic solvent extraction dramatically increased after litchi pericarp was treated with *Aspergillus*. The treatment of three fungi all significantly increased the phenolic contents both in the water fraction and ethyl acetate fraction. Since a large amount of polyphenols is conjugated to polysaccharide of cell walls or proteins in the cell membrane, it would be difficult to extract these polyphenols with aqueous-organic solvent [8]. Several hydrolytic enzymes produced by *A. awamori*, *A. sojae* and *A. oryzae* [18,19,20] likely released non-extractable polyphenols from litchi pericarp, thus increasing the extract recovery of the polyphenols. These hydrolytic enzymes may further hydrolyze glycosidic flavonoids to produce more hydrophobic aglycone in the ethyl-acetate fraction, which accounted for the enhanced DPPH radical scavenging activity of the fraction after the *Aspergillus* treatment. Furthermore, the hydrophobic products after the treatment of *A.* *awamori* exhibited higher DPPH radical scavenging activity than those treated with *A. oryzae* or *A. sojae*. Our previous study indicated that *A. awamori* had a great activity to cleave glycosidic bonds in rutin to form quercetin-3-glucoside and quercetin [15]. Additionally, the treatment of *Peronophythora litchii* with *Colletotrichum gloeosporioides* or *Fusarium proliferatum* was also conducted, which exhibited a high ability to increase the recovery of polyphenols from litchi pericarp, but with a low antioxidant activity (data not shown).

### 2.2. HPLC Analysis

**Figure 2:**
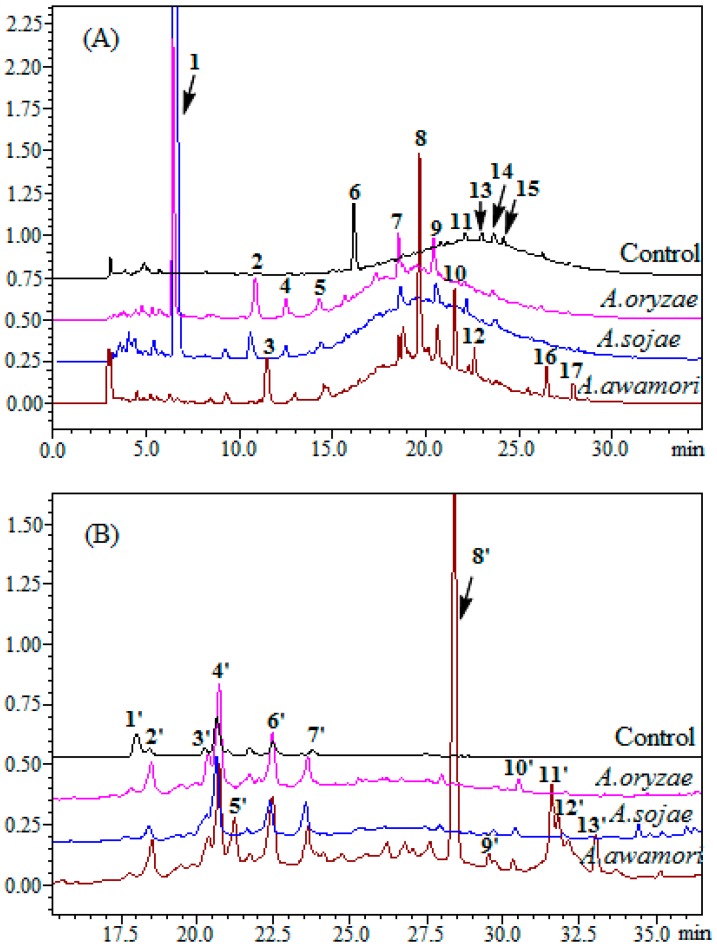
HPLC profile of hydrophilic (**A**) and hydrophobic (**B**) fractions of litchi pericarp from *A. oryzae*, *A. sojae* and *A. awamori* treatments.

The products of litchi pericarp after the treatment with *A. oryzae*, *A. sojae* or *A. awamori* were further analyzed by HPLC. The HPLC profiles are presented in Figure 2. As shown in the figure, Peaks 6, 11, 13, 14 and 15 were the major compounds present in the hydrophilic extract fraction of litchi pericarp without the fungus treatments, while Peaks 1’, 2’, 3’, 4’, 6’ and 7’ appeared in the hydrophobic extract fraction of litchi pericarp after the fungus treatment. In comparison with standard compounds, Peaks 6’, 11’, 7’ and 8’ were identified as procyanidin B2, epicatechin, -quercetin-3-glucoside and quercetin, respectively, which is consistent with the previous report [6,21]. The retention time of Peak 15 was matched with that of standard epicatechin-3-gallate, which is in agreement with the observation by Zhang et al. [21]. However, epicatechin-3-gallate was not detected in Sarni-Manchado’s report [6], likely because a different cultivar of litchi was used in these studies. The Huaizhi cultivar was used in Zhang’s study, while cultivar Kwai Mi was chosen in Sarni-Manchado’s. The same plant materials used in Zhang’s study were used in the present study. A large amount of compounds was eluted as a multi-overlapped peak at the retention time of 15–35 min, which is in agreement with the report by Roux et al. [22], who demonstrated the hump as condensed tannin with different degrees of polymerization. The area of Peak 6 decreased significantly after treatment with fungi, indicating that procyanidin B2 may be converted to other compounds. However, the area of Peak 11 (epicatechin) did not increase, possibly because procyanidin B2 was transformed to other flavonoids [23]. The HPLC profiles of the products of litchi pericarp after the treatment with *A. oryzae* were similar to that with the treatment of *A. sojae* (Peaks 1, 2, 4, 5, 7 and 9), indicating that *A. oryzae* and *A. sojae* may share similar enzymes [24].

### 2.3. The Effect of A. awamori Treatment on Condensed Tannin

**Figure 3:**
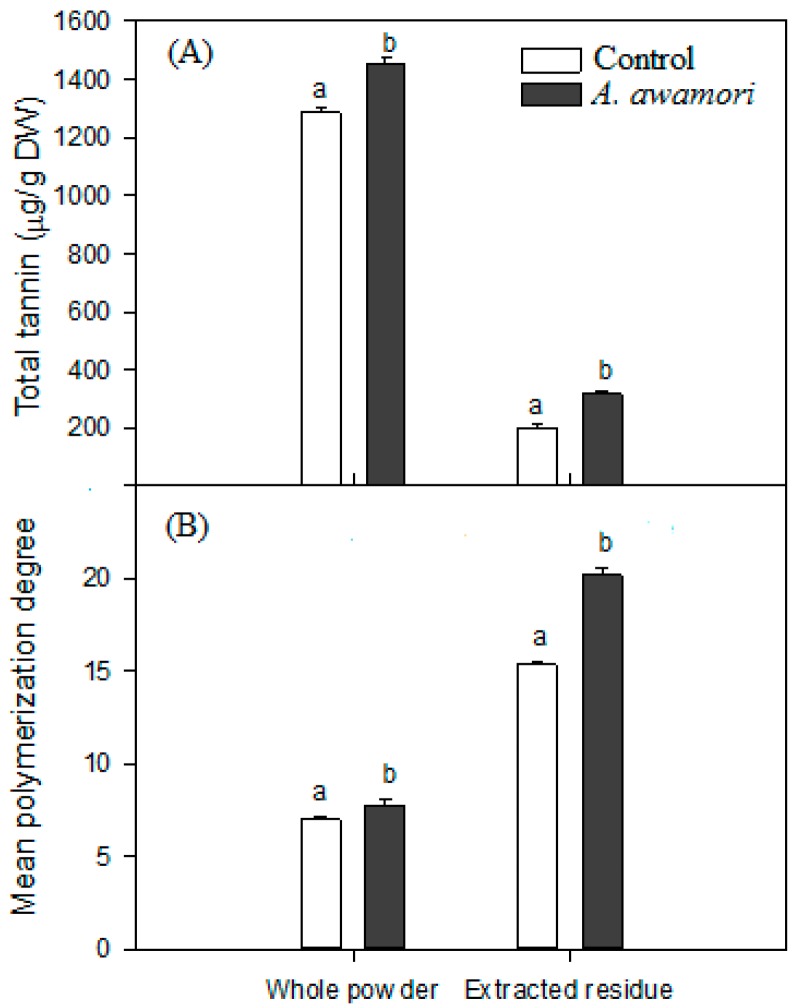
The changes in total tannin content (**A**) and the mean polymerization degree (**B**) present in litchi pericarp powder and the extracted residue after the *A. awamori* treatment. Data marked with the same letter above columns were not significant difference at *p* < 0.05 (mean ± SD, *n* = 3).

The contents of total tannin and the mean polymerization of tannin extracted from litchi pericarp after the *A. awamori* treatment were analyzed. To evaluate non-extractable condensed tannin, treatment of *A. awamori* on the extracted residue of litchi pericarp was also conducted. As shown in Figure 3, the content of total tannin increased significantly from 1286.58 ± 13.87–1456.12 ± 15.73 μg/g DW when whole litchi pericarp powder was treated with *A. awamori* and from 199.92 ± 14.47–318.38 ± 7.59 μg/g DW when the extracted litchi pericarp residue was treated with *A. awamori*. The mean polymerization degree also increased significantly after the treatment of *A. awamori*, suggesting that the high polymerized condensed tannin is bound to the cellular wall via a conjugation or physical force to form non-extractive condensed tannin [25,26].

### 2.4. ESI-TOF-MS

**Figure 4:**
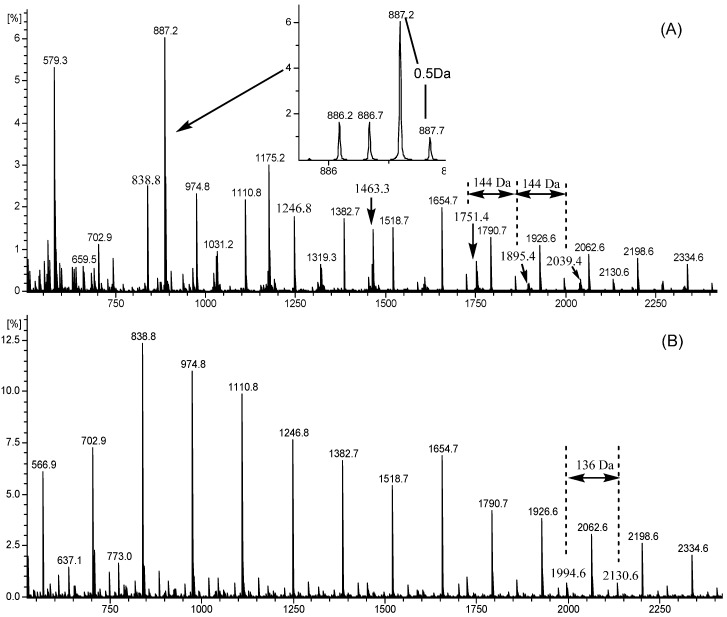
ESI-TOF-MS spectra of condensed tannin from LPP. (**A**) Control; and (**B**) *A. awamori*-treated condensed tannin.

Next, the products of litchi pericarp after the treatment of *A. awamori* were subjected to the ESI-TOF-MS analysis. The MS spectra are presented in Figure 4. Some peaks were doubly-charged molecules, as the difference of the nearby isotopic ion peak was 0.5 Da. After litchi pericarp was treated with *A. awamori*, many quasi-molecular ion peaks were missing, indicating the transformation of condensed tannin. Condensed tannins are oligomers and polymers of flavan-3-ols [8], and epicatechin is the major flavonoid in litchi pericarp [6,27]. These subunits may be condensed via a C4–C8 (B type) or C4–C8 coupled C2–O–C7 (A type) manner to form an oligomer or polymer. As the *m/z* value of the quasi-molecular ion was determined by the number of positive ion in each subunit, the number of each subunit can be estimated by comparing the calculated molecular weight with the *m/z* value observed from the MS spectra (Figure 4).

The *m*/*z* values for both *A. awamori*-treated and non-treated products obtained from the MS spectra and the proposed subunit are listed in Table 1. In this case, the polymerization degree of condensed tannin was 2–18 [27], and the major condensed tannin was the B-type epi/catechin hexamer (*m*/*z* 887.2 × 2), but epi/afzelechin and gallocatechin subunits were also observed. These results were somewhat different from the previous studies [28,29], possibly due to the different litchi cultivar used. Epi/afzelechin is a flavan-3-ol, which exhibits significant anti-inflammatory, analgesic and diuretic properties [30]. The ‘Huaizhi’ litchi employed in this study is the widest planted cultivar in south China. Our results indicated that the ‘Huaizhi’ cultivar can be used as the major source for epi/afzelechin. After the treatment of *A. awamori*, several peaks (*m*/*z* 579.3, 887.2, 1031.2, 1175.2, 1319.3, 1463.3 and 1751.4) disappeared, indicating the degradation of condensed tannin. Additionally, *A. awamori* treatment showed a high potential to degrade B-type condensed tannin (*m*/*z* 887.2), but a limited capacity to degrade those condensed tannin containing the A-type linkage subunit. The degradation of condensed tannins by gut microorganisms was reported previously [31,32]. It would be interesting to investigate the mechanism by which tannin is degraded by *Aspergillus*.

### 2.5. Phloroglucinolysis Analysis of Condensed Tannin

**Figure 5:**
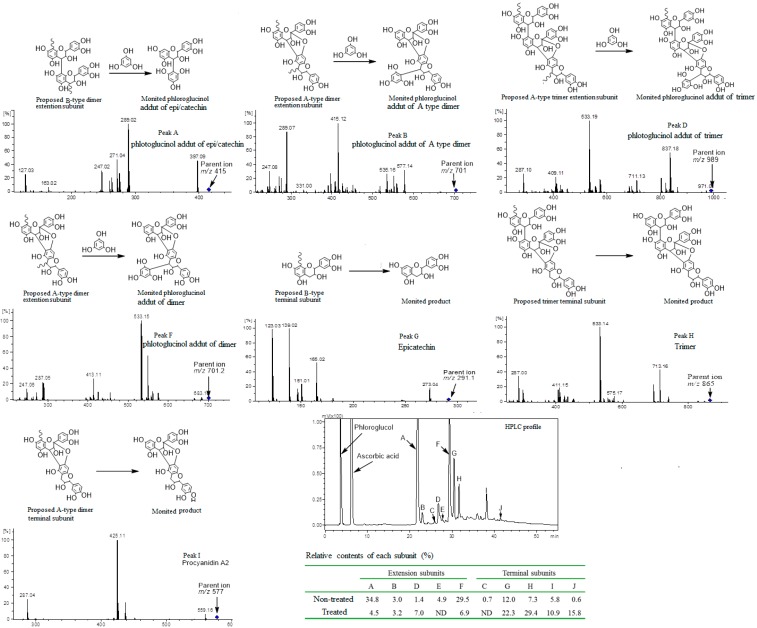
The structure changes of condensed tannin treated by *A. awamori.*

To further illustrate the effect of the treatment of *A. awamori* on condensed tannin, the product was subjected to HPLC-ESI-MS^2^ analysis. As shown in Figure 5, Compound A (Peak A in the HPLC chromatogram) possessed a quasi-molecular ion peak of *m*/*z* 415.1 [M + H]^+^ in positive mode and *m*/*z* 413.5 [M − H]^−^ and *m*/*z* 827.3 [2M − H]^−^ in negative mode. Thus, the molecular weight of Compound A was determined to be 414. Furthermore, Compound A has the MS^2^ peak of *m*/*z* 289.04 [M + H-phloroglucol]^+^, which was matched well with the neutral loss of the phloroglucinol moiety and subsequently identified as the phloroglucinol adduct of epicatechin [33,34]. Compound B, corresponding to Peak B in the HPLC chromatogram, possessed a quasi-molecular ion peak of *m*/*z* 701.5 [M + H]^+^ and MS^2^ peaks of *m*/*z* 577.1, 415.1 and 289.1 and was determined to be the phloroglucinol adduct of epi/catechin A-type dimer. Compound D, corresponding to Peak D in the HPLC chromatogram, had a quasi-molecular ion peak of *m*/*z* 989 [M + H]^+^ corresponding to a molecular weight of 988, and the MS^2^ fragment peak of *m*/*z* 837 [M + H − 152]^+^ resulting from the retro-Diels–Alder (RDA) fragmentation and the fragment [M + H − 152 − phloroglucinol]^+^ at *m*/*z* 711.3 corresponded to the elimination of a phloroglucinol molecule from the trimer. Thus, it was identified as the phloroglucinol adduct of the epi/catechin trimer containing both the A-type and B-type linkage, which indicated an extension subunit. Compound H had a quasi-molecular ion peak of *m/z* 865 [M + H]^+^ corresponding to a molecular weight of 864, and the MS^2^ fragment peak of *m/z* 713.1 [M + H − 152]^+^, resulting from the RDA fragmentation. This data indicated an epi/catechin trimer containing both the A-type and B-type linkage, which were previously reported as present in the litchi pericarp from the Kwai Mi cultivar [28] and consistent with the results of ESI-TOF observation (Table 1). However, those peaks observed in the MS^2^ spectra of Compound H could be contributed by the dehydrogenated products of the A-type dimer and an epi/catechin monomer. It is hard to differentiate between these two compounds with MS spectra because they had a similar chemical structure and shared many fragmentation ions in the MS^2^ spectra [35]. However, this dehydrogenated product was not reported in litchi pericarp before, while the epi/catechin trimer containing both the A-type and B-type linkage has been reported [28]. Thus, Compounds H and D are more likely to be epi/catechin trimers containing both the A-type and B-type linkage and its phloroglucinol adduct derivate, respectively, rather than the dehydrogenated products. Furthermore, Compounds E, F, G, I and J were determined to be the phloroglucinol adduct of afzelechin, the phloroglucinol adduct of the epi/catechin A-type dimer, epicatechin, the epi/catechin A-type dimer, procyanidin A2 and the epi/catechin A-type dimer, respectively [25,34]. These compounds of litchi pericarp were largely composed of epi/catechin with the A-type or B-type linkage. A minor afzelechin subunit was also detected. After the incubation, the proportion of extension subunits decreased while the terminal subunits increased. The B-type linked (C4–C8) moiety in condensed tannin (Peak A) was likely cleaved by *A. awamori* to produce terminal subunits (Peak G, epicatechin). The proportion of the A-type dimer extension subunit (Peak F) decreased while the A-type dimer terminal subunit (Peaks H, I and J) increased concurrently, possibly because *A. awamori* cannot degrade the A-type oligomer. In addition, the B-type linkage may be conversed to the A-type linkage by *laccase* [36] or radical oxidation [37], which may account for the increased A-type subunits.

## 3. Materials and Methods

### 3.1. Plant Materials and the Starter Microorganisms

Fresh fruit of litchi (*Litchi chinensis* Sonn.) cv. Huaizhi was harvested from a commercial orchard in Guangzhou, China. The fruit was peeled manually. The pericarp was collected, then dried in open air and finally ground into powder. *A. awamori*, *A. sojae* and *A. oryzae* were obtained from Guangdong Culture Collection Center, Guangzhou, China. After being cultured on potato dextrose agar at 30 °C for 3 days, the spores were collected by washing the agar surface with 20 mL of sterile distilled water containing 0.1% Tween 80. The spores were counted using a haemocytometer, and the spore suspension was prepared with a final concentration of ca. 10^6^/mL and served as inoculums for the further experiments.

### 3.2. Incubation of Aspergillus on Litchi Pericarp

Litchi pericarp powder (LPP, 25 g) was weighted into 500-mL flasks and then sterilized at 121 °C for 20 min. Fresh spore suspension (1 mL, ca. 10^6^ spores/mL) or the sterilized spore suspension was added into the flasks and then maintained at 30 °C for 6 days by stirring. The *Aspergillus*-treated LPP was extracted with 125 mL of 60% ethanol 3 times under the ultrasonic condition (210 W, 30 °C, 40 min). The ethanol was evaporated under vacuum at 50 °C, and then, the product was extracted two times with ethyl acetate (50 mL). The extractive phase was dried under vacuum and re-dissolved into 100 mL of ethanol. The affiant phase was evaporated to a final volume of 100 mL. To evaluate the effect of *Aspergillus* on the non-extractable condensed tannin, the incubation of *Aspergillus* with the LPP residue after 6 times of extraction with 60% ethanol was conducted. The total tannin content and mean polymerization degree of *Aspergillus*-treated and non-*Aspergillus*-treated LPP, as well as the LPP residue were comparatively analyzed. The experiments were conducted with triplicate biological repeats.

### 3.3. Incubation of Aspergillus with the Purified Condensed Tannin

To further reveal the change in the structure of condensed tannin after the application of *Aspergillus*, incubation on the purified condensed tannin was carried out. The litchi condensed tannin was extracted and purified with the following procures. LPP (50 g) was extracted 3 times using a solution of an acetone/water (70/30, *v*/*v*) mixture containing 0.1% vitamin C. After filtration, the acetone was removed under vacuum at 40 °C. The crude tannin was obtained after degreasing with 150 mL of n-hexane 3 times, and then, small molecular polyphenol was removed with 150 mL of ethyl acetate 3 times. The crude tannin was purified further with a Sephadex LH-20 column (1.5 cm × 100 cm). The column was then eluted with 50% methanol to remove sugars, followed by elution with an acetone/water (70/30, *v*/*v*) mixture. The elution solution was freeze dried and stored at −20 °C for further use. The incubation was conducted using 1 mL of fresh/sterilized spore suspension of *A. awamori* incubated with 20 mL of the sterilized Czapek medium (121 °C, 30 min) containing 0.15 g of NaNO_3_, 0.02 g of K_2_HPO_4_, 0.01 g of KCl, 0.01 g of MgSO_4_·7H_2_O, 0.2 mg of FeSO_4_ and 3 g of sucrose for 2 days at 28 °C at 180 rpm rotation. Twelve milligrams of the purified condensed tannin were then added and cultured for another 4 days. The products were freeze dried and then extracted with acetone/water (70/30, *v*/*v*) before analysis. The experiments were conducted with triplicate biological repeats.

### 3.4. Measurements of Contents of Total Phenolics and Flavonoids

The content of total phenolics was determined according to the method of Quettier et al. [38]. In brief, samples (0.1 mL) were mixed with 5 mL of distilled water and 0.5 mL of Folin–Ciocalteu phenol reagent and incubated for 8 min at room temperature. Twenty percent Na_2_CO_3_ (1.5 mL) was then added and heated in boiling water for 1 min. The absorbance was recorded at 760 nm using a spectrophotometer (Uico Shanghai Instrument Co., Ltd., Shanghai, China). Total phenolic content was expressed as milligrams of gallic acid equivalent (GAE) on a dry weight (DW) basis (mg GAE/g DW) from the calibration curve of the standard gallic acid. Total flavonoid content was determined by a colorimetric method using rutin as a reference compound [39]. Samples (1 mL) were mixed with 4 mL of distilled water and 0.5 mL of 5% sodium nitrite. After incubation of 6 min, 0.5 mL of 10% aluminum nitrate was added and then allowed to stand for another 5 min before 4 mL of 1 M NaOH were added. The absorbance at 510 nm was measured immediately against 60% ethanol as a blank. Total flavonoid content was expressed as rutin equivalent (RE) on a dry weight basis (mg RE/g DW). All of the tests were conducted in triplicate.

### 3.5. HPLC Analysis

Samples were analyzed with a Shimadzu LC-20 AT HPLC (Shimadzu Corporation, Kyoto, Japan) coupled with a Vydac C18 column (218 TP, 250 mm × 4.6 mm, 5-μm particle size, Sigma-Aldrich, St. Louis, MO, USA) and an SPD-10A UV–VIS detector. Solvents A (0.1% trifluoroacetic acid) and B (methanol) were used as the mobile phases at a flow rate of 1 mL/min. The gradient elution was conducted by the method of Yang and Zhai [40]: 0–5 min (10% B), 5–35 min (10%–100% B), 35–40 min (100% B) and 40–45 min (10% B). The injection volume was 20 μL. The chromatogram with the retention time was recorded at 280 nm.

### 3.6. DPPH Radical Scavenging Assay

The extracts of the *Aspergillus*-treated and non-*Aspergillus*-treated LPP were subjected to the assessment of DPPH radical scavenging activity [41]. The control was carried out with 60% ethanol instead of the tested sample, whilst methanol instead of DPPH was used as a blank. The DPPH radical scavenging activity of the tested sample was calculated as the following equation.

(1) $$\text{DPPH radical scavenging activity }\left(\text{\%}\right)=(1-\frac{\text{absorbance of sample}-\text{absorbance of blank}}{\text{absorbance of control}}>)\times 100$$

### 3.7. Measurements of Total Tannin and Mean Polymerization Degree

The total tannin content was measured with the Bate-Smith method [40]. The *A. awamori*-treated or non-*A. awamori*-treated condensed tannin liquid was dried under vacuum and re-dissolved into methanol before 3 mL of n-butyl alcohol-HCl (95/5, *v*/*v*) were added. After 40 min of reaction at 97 °C, the mixture was rapidly cooled with cold water. Methanol instead of the tested sample was employed as the blank. The purified condensed tannin with a purity of 92% was employed as the reference compound. Total tannin content was quantified with a standard curve (*Y* = 307.02*X* + 2.6701, *R*^2^ = 0.9972), where *Y* and *X* were the concentration of the condensed tannin (μg/mL) and the optical absorbance at 550 nm, respectively.

The terminal residue unit of the condensed tannin was determined by the method previously described by Butler et al. [4]. In brief, 0.5 mL of samples were mixed with 3 mL of vanillic aldehyde-glacial acetic acid (4%, *v*/*v*) solution and 1.5 mL of 4% HCl, followed by reaction for 5 min at 20 °C. Methanol instead of sample was used as a blank. The absorbance at 500 nm was recorded with a spectrophotometer. The amount of terminal catechol was determined by a colorimetric method using catechol as a reference with the standard curve (*Y* = 315.42*X* − 1.6001, *R*^2^ = 0.9986), where *Y* and *X* were the concentration of the terminal unit (μg/mL) and the optical absorbance at 500 nm, respectively. The mean polymerization degree was calculated as the ratio of total tannin to the amount of terminal residue.

### 3.8. ESI-TOF-MS Analysis

To further understand the effect of incubation on the molecular weight of the condensed tannin, the *A. awamori*-treated or non-*A. awamori*-treated condensed tannin was subjected to ESI-TOF-MS analysis. Electrospray time-of-flight mass spectra (ESI-TOF-MS) (accurate mass) were measured in the positive-ion mode using an Agilent MSD-TOF mass spectrometer with a capillary voltage of 3000 V, nebulizer pressure of 40 psi, drying gas of 4 L/min and mass range of *m*/*z* 100–3500. Samples from three parallel incubations described in Section 3.4 were analyzed.

### 3.9. Phloroglucinolysis Analysis of Condensed Tannin

**Figure 6:**
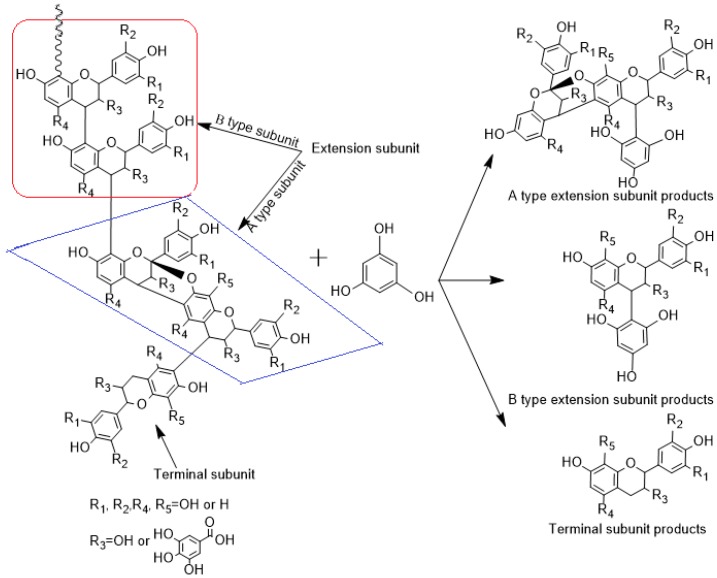
Nucleophilic reaction of condensed tannin and phloroglucol.

Condensed tannin is a kind of large molecular polyphenol composed of several kinds of flavan-3-ol, including epi/catechin, epi/afzelechin or their gallate derivants with the C4–C8 linkage (B-type) or the C4–C8 coupled with C2–O–C7 double linkage (A-type) [42,43]. The C4 position of the B-type linkage in the condensed tannin can be attacked with phloroglucinol to form a free terminal subunit and phloroglucinol adduct of extend subunits (A-type or B-type) (Figure 6). The released terminal subunit and phloroglucinol adduct of extension subunits can be identified with HPLC-ESI-MS^2^. Briefly, the condensed tannin (5 g/L) was reacted for 20 min with a solution of 0.1 M HCl in MeOH, containing 50 g/L phloroglucinol and 10 g/L of ascorbic acid at 50 °C, and the reaction was stopped by the addition of 5 volumes of 40 mM aqueous sodium acetate [44].

The products were identified using an Agilent 1100 series HPLC system (Agilent Technologies, Waldbronn, Germany) coupled with a Vydac C18 column and a diode array detector (DAD) and an ion-trap mass spectrometer fitted with an ESI source (Bruker Daltonics HCT Ultra, Bremen, Germany). Solvents A (0.1% formic acid) and B (acetonitrile) were used as the mobile phase at a flow rate of 0.8 mL/min, with the following gradient elution program of 5% B for 0–10 min, 5%–20% B for 10–30 min, 40% B for 30–55 min and 100% B for 55–60 min. The temperature of the column was maintained at 25 °C. The injection volume was 20 μL. The MS was operated in both positive and negative modes with Auto-MS^n^ using a ramping of the collision energy. The operation condition was set as follows: capillary voltage of 4000 V and skimmer voltage of 30 V. The nebulizer pressure was 40 psi at a nitrogen flow rate of 9 L/min. The monitored mass range was from *m*/*z* 70–1200. Three parallel incubations described in Section 3.4 were used for phloroglucinolysis analysis of condensed tannin.

### 3.10. Statistical Analysis

Data are expressed as the means ± standard deviations (SD) and then were analyzed by OriginPro 8 (OriginLab Corporation, Northampton, MA, USA). One-way analysis of variance (ANOVA) and Tukey’s multiple comparisons were carried out to test any significant differences between the means. Differences between means at the 5% level were considered significant.

## 4. Conclusions

In this study, the effects of *A. awamori*, *A. sojae* and *A. oryzae* incubations with LPP on polyphenol extraction recovery and DPPH radical scavenging activity of litchi pericarp were investigated. The study suggested that these incubations can increase significantly the extraction recovery of polyphenols from litchi pericarp, possibly because of the release of non-extractable condensed tannin. Furthermore, the products from the application of *A. awamori* possessed the highest antioxidant activity among them. It was found that *A. awamori* treatment can degrade B-type condensed tannin (condensed flavan-3-ol via the C4–C8 linkage) well, but had a low capacity to degrade the condensed tannin containing many A-type linkage subunits (C4–C8 coupled C2–O–C7 linkage). These results suggest that application of *A. awamori* can significantly improve the production of condensed tannin from litchi pericarp.

## Figures and Tables

**Table 1:** The proposed subunits of litchi pericarp condensed tannin and the changes after *A. awamori* incubation with condensed tannin.

Polymers	Proposed Ionization Type			Charge (CH)	A Type Linkage (AL)	Proposed Subunit			Calculated Masses * (*m*/*z*)	Observed Masses (*m*/*z*)	
	H	Na	K			Af	Ca	Ga		NT	T
1-mers	1	0	0	1	0	0	2	0	579	579.3	-
3-mers	0	1	0	1	0	3	0	0	838	838.8	838.8
	1	0	0	1	1	0	3	0	865	865.2	865.2
	0	1	0	1	0	0	2	1	903	903.2	-
	0	1	0	0	1	0	0	3	937	937.4	-
4-mers	0	1	0	0	1	4	0	0	1110	1110.8	1110.8
	0	1	0	1	1	0	0	4	1382	1382.7	1382.7
5-mers	0	0	1	1	0	0	5	0	1479	1479.3	-
	1	0	0	1	1	0	0	5	1518	1518.7	1518.7
6-mers	0	2	0	2	1	0	6	0	887	887.2	-
	0	1	1	2	2	0	6	0	894	894.2	-
	0	0	2	0	0	0	6	0	954	954.2	-
	0	1	0	1	0	6	0	0	1654	1654.7	1654.7
	1	0	0	1	1	0	2	4	1790	1790.7	1790.7
	1	0	0	1	2	0	3	3	1926	1926.6	1926.6
7-mers	2	0	0	2	2	0	3	4	1039	1039.2	-
	0	2	0	2	0	0	7	0	1183	1183.2	-
	1	0	0	1	2	0	4	3	2062	2062.6	2062.6
8-mers	0	2	0	2	1	0	6	2	1191	1191.2	-
9-mers	2	0	0	2	1	0	6	3	1319	1319.3	-
	0	1	1	2	0	0	9	0	1327	1327.3	-
	0	2	0	2	0	1	9	0	1450	1450.7	1450.7
10-mers	0	2	0	2	1	0	10	0	1463	1463.3	-
	0	2	0	2	1	0	9	1	1471	1471.3	-
11-mers	0	2	0	2	1	2	9	0	1586	1586.7	1586.7
	0	2	0	2	1	0	9	2	1615	1615.3	-
12-mers	0	2	0	2	1	3	9	0	1722	1722.7	1722.7
	0	2	0	2	1	0	12	0	1751	1751.4	-
13-mers	0	2	0	2	1	4	9	0	1858	1858.7	1858.7
	0	2	0	2	1	0	13	0	1895	1895.4	-
14-mers	0	2	0	2	1	5	9	0	1994	1994.6	1994.6
	0	2	0	2	1	0	14	0	2039	2039.4	-
15-mers	0	2	0	2	1	6	9	0	2130	2130.6	2130.6
	0	2	0	2	1	0	15	0	2183	2183.5	-
16-mers	0	2	0	2	1	7	9	0	2266	2266.6	2266.6
	0	2	0	2	1	0	16	0	2327	2327.5	-
17-mers	0	2	0	2	1	8	9	0	2402	2402.6	2402.6

* The molecular weight of the proposed quasi-molecular ion peaks can be calculated with the following equation: molecular weight = CH (2 + H + 23Na + 39K + 272Af + 288Ca + 304Ga − 2AL). CH is the charge of quasi-molecular ion, and AL and GS stand for the number of A-type linkage subunits and the number of gallate flavan-3-ol subunits, while Af, Ca and Ga are the number of epi/afzelechin, epi/catechin and epi/gallocatechin subunits, and T and NT mean *A. awamori*-treated and non-*A. awamori*-treated samples, respectively.

## Abbreviations

LPP	Litchi pericarp powder
DPPH	2,2-Diphenyl-1-picrylhydrazyl
DW	Dry weight
GAE	Gallic acid equivalent
RE	Rutin equivalent
HPLC-MS	High performance liquid chromatography electrospray-mass spectrometer
ESI-TOF-MS	Electrospray ionization-time of flight-mass spectrometry
